# Comments on “Impact of climate change on dentistry and oral health: a scoping review”

**DOI:** 10.1038/s41405-025-00375-z

**Published:** 2026-01-05

**Authors:** Gopika Mohanakumaran Nair Geetha, Raj Mohan

**Affiliations:** 1 Independent Researcher, Sheffield, UK; 2 Respiratory Department, General Practitioner (GP) Registrar, Northern General Hospital, Sheffield, UK

**Keywords:** Xerostomia, Dental epidemiology

We read with great interest the recent article by Bhadauria et al., titled “Impact of Climate Change on Dentistry and Oral Health: A Scoping Review” [1]. The authors have taken an important step in synthesizing literature on a subject of growing concern, and their effort to highlight the multifaceted ways in which climate change could influence oral health is commendable.

While we appreciate the scope and intent of the review, we would like to respectfully raise the following points for clarification and further consideration:

1. **Reference accuracy**
  We noted a citation error involving references 9 and 10. Both entries are cited as “Ajit SL. Effects of Climate Changes on Oral Health” and link to the same editorial article. However, Table 1 in the review clearly differentiates two distinct publications attributed to an author of the same name: an editorial [2] and a commentary titled “Effects of Global Climate Change on Oral Health: Overview” [3]. The second citation should be updated to reflect the correct source for accuracy and consistency. In addition, the year listed in Table 1 for the editorial should be corrected to 2021 rather than 2020, to align with the original publication details.
2. **Inclusion criteria and use of the Grose et al. study**
  The review outlines clear inclusion and exclusion criteria, stating that only studies evaluating the impact of climate change on oral health and dentistry were eligible for inclusion [1]. One of the included studies, “Exploring attitudes and knowledge of climate change and sustainability in a dental practice: A feasibility study into resource management” by Grose et al., appears to focus primarily on how dental practices contribute to environmental impact, rather than on how climate change affects oral health. Although the article mentions climate change and sustainability, it does not explore any direct or indirect effects of climate change on oral health outcomes. Its inclusion may therefore be inconsistent with the stated criteria of the review. Additionally, the review cites Grose et al. as supporting the claim that climate change will negatively affect health service provision and lead to resource shortages due to extreme weather events. These assertions, however, are only found in the “In Brief” editorial summary of the article and are not supported or discussed in the main text [4]. As such, using this article as evidence for climate-related health service disruptions could be misleading.
3. **Epidemiological evidence**
  The authors acknowledge that “There is a lack of epidemiological studies that can provide deeper insights into the long-term impacts of climate change on oral health”, identifying this as a significant gap in the literature [1]. While broad gaps remain, several areas already provide substantial and well-established evidence. Biomedical studies of ultraviolet (UV) radiation, for instance, have consistently demonstrated a causal relationship between increased solar exposure (a climate-related factor) and oral malignancies, particularly lip cancer [5].
  Similarly, we would like to highlight nationwide, population-based studies from Taiwan and China, which demonstrated a strong association between prolonged exposure to air pollution, a climate-related environmental factor, and increased risk of periodontal disease [6, 7]. These studies provide robust epidemiological evidence, enhancing our understanding of the environmental determinants of oral health and bridging existing knowledge gaps.
4. **Recommendations**
  Finally, we note that the article does not discuss the integration of sustainability into dental education or the development of updated clinical guidelines that support environmentally responsible dental practice. Given the importance of preparing future dental professionals to meet environmental challenges, the absence of commentary on curriculum reform or eco-friendly practice guidelines under the review’s “intervention strategies” section represents a missed opportunity. Encouraging the incorporation of climate-conscious principles into dental training and professional standards could be a valuable area for future exploration.

In conclusion, the authors have made a valuable contribution to an emerging field. We hope these additional observations will enrich the discussion on the intersection of climate change, dentistry, and public health.
